# Supplementary material for: The allergic response mediated by fire ant venom proteins
Source: Sci Rep. 2018 Sep 26;8:14427. doi: 10.1038/s41598-018-32327-z (PMC6158280; doi:10.1038/s41598-018-32327-z)
Supplement: Supplementary file 1 — Supplementary Figures [file 41598_2018_32327_MOESM1_ESM.docx]

**The allergic response mediated by fire ant venom proteins**

Daniel Zamith-Miranda^ф1^, Eduardo G. P. Fox ^ф2^, Ana Paula Monteiro^1^, Diogo Gama^3^, Luiz E. Poublan^1^, Almair Ferreira de Araujo^1^, Maria F. C. Araujo^4^, Georgia C. Atella^4^, Ednildo A. Machado^3^, Bruno L. Diaz^*1^

^ф^ Both authors have contributed equally to this project

* Correspondence to bldiaz@biof.ufrj.br

**Supplementary Information**


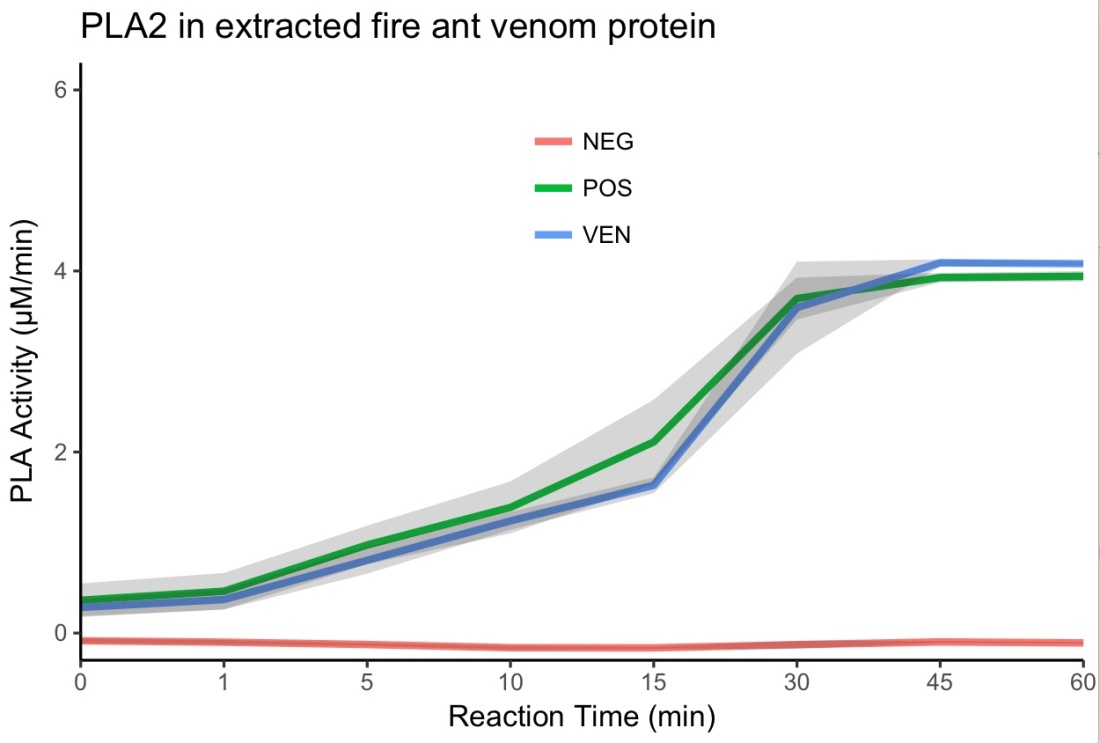


Figure S1. **Phospholipase A_2_ activity from a venom protein extract of the fire ant** *Solenopsis invicta*. Venom protein was obtained as in Fox et al. (2013). Samples were assayed for PLA_2_ activity using the fluorescent substrate NBD-PC with 10 μg of fire ant venom protein (VEN, in blue), positive control (13 µg of *Bothrops jararaca* venom; POS, in green) and negative control group (NEG, in red). Lines represent means; shaded area are standard errors measured at each analysis time point (N = 3 replicates per group). Activity from VEN was statistically similar to POS (W = 351.5, p-value = 0.8288), and significantly different from NEG (W = 43.5, p-value = 2.94e-08) by non-parametric paired Wilcoxon-Mann-Whitney test.


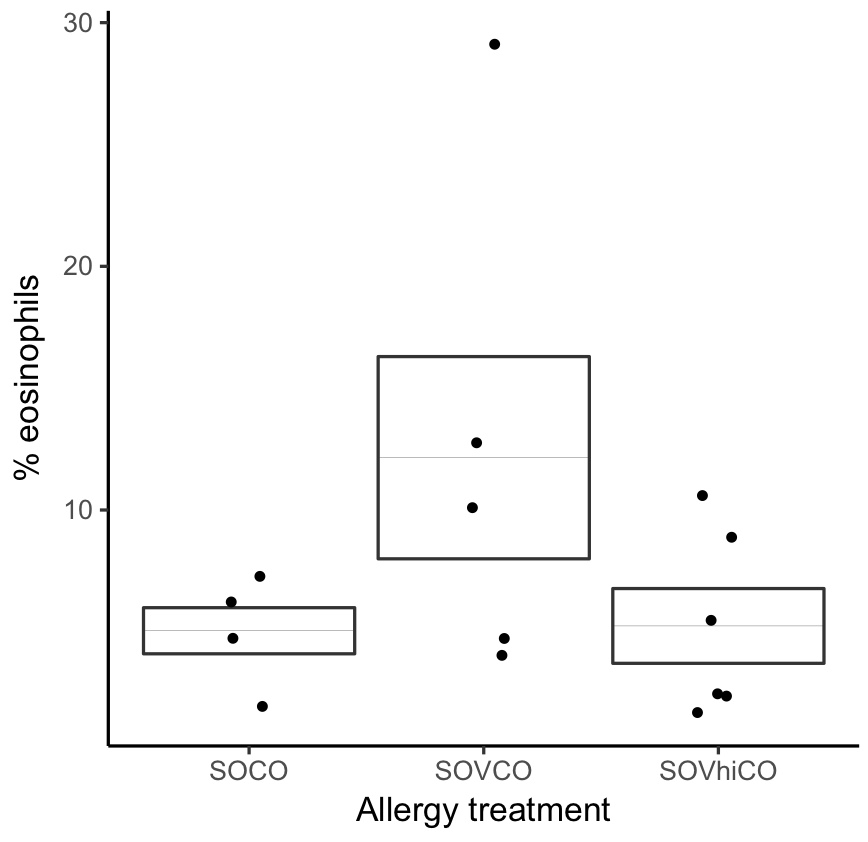


Figure S2. **Boiling fire ant venom proteins inactivates adjuvant properties.** Venom protein was obtained as in Fox et al. (2013). Mice were previously sensitized with 100 µg ovalbumin alone (SOCO), ovalbumin (OVA) + 10 µg RIFA venom proteins (SOVCO), or ovalbumin (OVA) + 10 µg RIFA venom proteins which were boiled for 60 min at 100oC (SOVhiCO). After 2 weeks mice were respectively challenged with another exposure to saline, . Cell counts recovered from peritoneum are represented above for each treatment (dots), where a box represents means (internal line) ± SE (upper and lower limits). SOVhiCO cell counts were statistically equivalent to saline SOCO controls by nonparametric Wilcoxon-Mann-Whitney at alpha = 0.05 (W = 21, p-value = 0.6991).
