# Supplementary material for: The allergic response mediated by fire ant venom proteins
Source: Sci Rep. 2018 Sep 26;8:14427. doi: 10.1038/s41598-018-32327-z (PMC6158280; doi:10.1038/s41598-018-32327-z)
Supplement: Supplementary file 2 — Raw Data [file 41598_2018_32327_MOESM2_ESM.pdf]

Supplementary containing Raw Data for the paper "The allergic response mediated by fire ant venom proteins" by Zamith-Miranda et al. 2018. Information on Methods can be found on the paper narrative. Usually independent experiments were made with internal replicates, with total N depending on available number of animals in good shape for experimentation. *************************   Data recovered from Prism 7 files using OCR recognition of captured screen ********************

#Eosinophils % recruitment to peritoneal cavity following antigen exposure

Saline_1
3.000000
1.333333
0.3333333

Saline_2
0.000000
0.000000
2.000000

Saline_3
1.000000
0.000000

SOACO_1
9.000000
28.333330
13.333330

SOACO_2
18.000000
29.000000
22.000000

SOACO_3
1.000000
22.000000

S10C10_1
3.666667
4.666667
9.666667
9.333333

S10C10_2
11.000000
13.000000
16.000000

S10C10_3
17.000000
16.000000
14.000000

S100C10_1

10.000000
7.333334
7.000000
5.000000

S100C10_2
20.000000
15.000000
14.000000

S100C10_3
19.000000
15.000000

Sal/C10_1
2.00
3.00
12.00

Sal/C10_2
5.00

SOVCO_1
10.00
17.00
23.00

SOVCO_2
13.00
9.00

#Eotaxin(pg/mL)

Saline_1
136.00
187.00

Saline_2
300.00

SOACO_1
609.00

SOACO_2
664.00

S100C10_1
366.00
457.00

S100C10_2
812.00
597.00

#DC MHCII/CD86

Ctr-_1
18.9
15.3
18.4
16.5

Ctr-_2
10.1
11.9

Ctr-_3
8.6
6.9

Ctr+_1
26.88
27.20
27.75
32.50

Ctr+_2
12.50
18.11

Ctr+_3
11.90
9.40

V100_1
23.70
19.50
21.80
16.50

V100_2
15.05
20.60

V100_3
10.15
7.30

V10_1
26.0
21.4
19.6
18.7

V10_2
13.7
18.9

V10_3
8.4
7.3

V1_1
26.30
21.80
19.40
22.90

V1_2
17.00
20.20

V1_3
9.66
7.70

V0.1_1
26.50
23.00
25.73
24.90

V0.1_2
16.50
19.30

V0.1_3
10.60
7.40

#DC MHCII

WT-_1
22.90
19.00
21.90
20.40

WT-_2
12.80
15.00

WT-_3
12.50
9.40

Ctr+_1
23.93
26.11
25.95
29.50

Ctr+_2
13.80
20.20

Ctr+_3
14.80

12.10

WT Ven 100_1
27.20
23.80
24.20
19.10

WT Ven 100_2
19.30
24.20

WT Ven 100_3
11.53
7.90

WT Ven 10_1
30.30
26.20
22.10
22.40

WT Ven 10_2
16.30
22.20

WT Ven 10_3
11.10
9.90

WT Ven 1_1
30.10
26.00
23.30
26.80

WT Ven 1_2
20.20
23.70

WT Ven 1_3
13.30
9.80

WT Ven 0.1_1
30.60
27.60
29.30
29.10

WT Ven 0.1_2
19.70
22.50

WT Ven 0.1_3
14.10

9.40

#DC CD86

Ctr-_1
26.5
20.8
26.6
22.9

Ctr-_2
14.7
17.3

Ctr-_3
26.0
22.9

Ctr+_1
45.975
46.150
44.750
49.000

Ctr+_2
16.900
23.500

Ctr+_3
54.000
53.000

V100_1
31.20
27.60
30.30
25.40

V100_2
22.15
26.70

V100_3
28.70
21.70

V10_1
33.90
26.60
31.10
28.80

V10_2
19.30
25.30

V10_3
24.60
22.40

V1_1
35.80
26.50
30.10
31.00

V1_2
23.30
25.70

V1_3
27.40
25.50

V0.1_1
34.16
27.90
35.80
33.20

V0.1_2
22.60
24.70

V0.1_3
32.30
23.90

#Footpad swelling
#Main compilation

Treat Saline1 Saline2 Saline3
Inoculated 3.603333 5.470667 4.345
30m 2.93 4.024 3.685
60m 1.006667 3.987333 2.9
90m 0.6066667 2.650667 2.735
120m -0.1033333 0.964 1.47

Treat SOACO1 SOACO2 SOACO3
Inoculated 3.95 5.323333 5.383333
30m 6.25 8.41 5.283333
60m 4.37 7.426667 4.743333
90m 4.13 7.803333 4.203333
120m 2.513333 5.036667 4.036667

Treat S10C10a S10C10b S10C10c
Inoculated 4.685 5.486667 4.755
30m 4.755 7.473333 4.906667
60m 3.9425 6.753334 4.263333
90m 3.345 4.7 4.253333

120m 0.325 3.826667 4.806667

#Demonstrating self-contained adjuvancy

Treat SOVCO1 SOVCO2
Inoculated 5.373333 4.615
30m 6.38 4.77
60m 5.03 4.36
90m 4.346667 3.59
120m 2.583333 2.705

Treat SOCO1 SOCO2
Inoculated 5.666667 4.135
30m 4.153333 2.52
60m 3.48 2.975
90m 2.36
120m 0.336667 2.21

#Cels/LN

Saline_1
0.426000
1.858000

Saline_2
0.275000

Saline_3
1.250000

SOACO_1
13.730000
17.290000
16.125000

SOACO_2
22.250000
18.250000

SOACO_3
16.400000

S10_1
1.670000
2.250000
4.500000

S10_2
4.250000
1.250000

S10_3
2.070000

S100_1
1.060000
3.750000
3.500000

S100_2
2.125000
0.625000

S100_3
3.275000

#IL-4 LN Cells

Saline_24h
0
0

Saline_48h
0
0

OVA_24h
25.2
27.4

OVA_48h
13.5
25.6

S10_24h
13.3
15.8

S10_48h
25.5
23.0

S100_24h
6.3
9.3

S100_48h
17.2
13.0

#Supplementary Experiment on cooking venom proteins, to test for inactivation of enzymatic activity
#Eosinophils%
OVA/OVA
7.278481
1.941748
4.733728
6.227106

OVA+V/OVA
29.113920
10.096150
12.757200
4.729730
4.035874
2.400000*

OVA+V(HI)/OVA
10.594320
8.881579
5.472637
1.687764
2.362205
2.459016

#Total eosinophils
OVA/OVA
0.509494
0.180583
0.411834
0.759707

OVA+VIOVA
3.144304
0.696635
0.816461
0.156081
0.339014
0.194400

OVA+V(HI)/OVA
1.080620
0.559540
0.268159
0.175527
0.240945
0.255738

#Supplementary experiment demonstrating PLA2 activity
#Negative control
min 0 1 5 10 15 30 45 60
1 -0.104240 -0.119720 -0.130640 -0.148020 -0.140860 -0.071340 -0.089520 -0.104080
2 -0.038120 -0.053190 -0.097620 -0.136540 -0.173910 -0.213210 -0.085910 -0.093390
3 -0.113260 -0.129190 -0.151170 -0.201010 -0.175960 -0.100360 -0.118150 -0.129300

#Positive control
min 0 1 5 10 15 30 45 60
1 0.336392 0.448064 1.228570 1.749530 2.982712 4.024175 4.025494 4.036731
2 0.055261 0.122334 0.550693 0.820465 1.385042 3.248932 3.907618 3.952554
3 0.696089 0.818258 1.141122 1.593919 1.965230 3.812742 3.849037 3.834301

#Venom

min	0	1	5	10	15	30	45	60
1	0.149357	0.213174	0.600795	1.111205	1.797345	4.145456	4.154486	4.121151
2	0.465277	0.584700	1.091691	1.432507	1.598656	2.580273	4.021995	4.044093
3	0.232876	0.314039	0.719452	1.174039	1.499796	4.058950	4.095057	4.077280
