# Supplementary material for: The allergic response mediated by fire ant venom proteins
Source: Sci Rep. 2018 Sep 26;8:14427. doi: 10.1038/s41598-018-32327-z (PMC6158280; doi:10.1038/s41598-018-32327-z)
Supplement: Supplementary file 3 — Data Analysis and Plotting RScript [file 41598_2018_32327_MOESM3_ESM.pdf]

```
#*************Script for Data Analysis and plotting written by Eduardo G P
Fox for the Paper *****************************

#                The allergic response mediated by fire ant venom proteins
#            by Zamith et al. 2018


#Firstly making sure R workspace is clean for correct data input and
montage
rm(list = ls())


#Loading useful packages
require(plyr)
require(reshape2)
require(ggplot2)
require(miscTools)
require(conover.test)
require(gplots) # just for the legends function

#adding useful functions from other sources
#From The R book by Michael J. Crawley, 2ed. ISBN 978-0-470-97392-9
se <- function(x) sqrt(var(x)/length(x))
ci95 <- function(x) {
t.value <- qt(0.975,length(x)-1)
standard.error <- se(x)
ci <- t.value*standard.error
cat("95 Confidence Interval = ", mean(x) -ci, "to ", mean(x) +ci," \ n") }

#from the website "R cookbook" @ cookbook-
r.com/Graphs/Multiple_graphs_on_one_page_(ggplot2)/
multiplot <- function(..., plotlist=NULL, file, cols=1, layout=NULL) {
  library(grid)

  # Make a list from the ... arguments and plotlist
  plots <- c(list(...), plotlist)

  numPlots = length(plots)

  # If layout is NULL, then use 'cols' to determine layout
  if (is.null(layout)) {
    # Make the panel
    # ncol: Number of columns of plots
    # nrow: Number of rows needed, calculated from # of cols
    layout <- matrix(seq(1, cols * ceiling(numPlots/cols)),
                    ncol = cols, nrow = ceiling(numPlots/cols))
  }

 if (numPlots==1) {
    print(plots[[1]])

  } else {
    # Set up the page
    grid.newpage()
    pushViewport(viewport(layout = grid.layout(nrow(layout),
ncol(layout))))

    # Make each plot, in the correct location
```

```
    for (i in 1:numPlots) {
      # Get the i,j matrix positions of the regions that contain this
subplot
      matchidx <- as.data.frame(which(layout == i, arr.ind = TRUE))

      print(plots[[i]], vp = viewport(layout.pos.row = matchidx$row,
                                      layout.pos.col = matchidx$col))
    }
  }
}

#For making a blank column later
insertRow <- function(existingDF, newrow, r) {#some genius function taken
from Stackoverflow
  existingDF[seq(r+1,nrow(existingDF)+1),] <-
existingDF[seq(r,nrow(existingDF)),]
  existingDF[r,] <- newrow
  existingDF
}


#NA-filled data.frame build, obtained from
https://stackoverflow.com/a/7196683/7332987
na.pad <- function(x,len){
    x[1:len]
}

Filled.DataFrame <- function(l,...){
    maxlen <- max(sapply(l,length))
    data.frame(lapply(l,na.pad,len=maxlen),...)
}

#Now all is set for starting
*****************************************************

#Importing Data from separate raw data file
#Eosinophils recruitment to peritoneal cavity following antigen exposure
EosinophilsSaline1<-
scan("/Users/egoncal2/Work_Files/Data/Scripts/data_camundongos.txt",
skip=7, nlines=3)
EosinophilsSaline2<-
scan("/Users/egoncal2/Work_Files/Data/Scripts/data_camundongos.txt",
skip=12, nlines=3)
EosinophilsSaline3<-
scan("/Users/egoncal2/Work_Files/Data/Scripts/data_camundongos.txt",
skip=17, nlines=2)
EosinophilsSOACO1<-
scan("/Users/egoncal2/Work_Files/Data/Scripts/data_camundongos.txt",
skip=21, nlines=3)
EosinophilsSOACO2<-
scan("/Users/egoncal2/Work_Files/Data/Scripts/data_camundongos.txt",
skip=26, nlines=3)
EosinophilsSOACO3<-
scan("/Users/egoncal2/Work_Files/Data/Scripts/data_camundongos.txt",
skip=31, nlines=2)
EosinophilsS10C10_1<-
scan("/Users/egoncal2/Work_Files/Data/Scripts/data_camundongos.txt",
skip=35, nlines=4)
EosinophilsS10C10_2<-
scan("/Users/egoncal2/Work_Files/Data/Scripts/data_camundongos.txt",
```

```
skip=41, nlines=3)
EosinophilsS10C10_3<-
scan("/Users/egoncal2/Work_Files/Data/Scripts/data_camundongos.txt",
skip=46, nlines=3)
EosinophilsS100C10_1<-
scan("/Users/egoncal2/Work_Files/Data/Scripts/data_camundongos.txt",
skip=51, nlines=4)
EosinophilsS100C10_2<-
scan("/Users/egoncal2/Work_Files/Data/Scripts/data_camundongos.txt",
skip=57, nlines=3)
EosinophilsS100C10_3<-
scan("/Users/egoncal2/Work_Files/Data/Scripts/data_camundongos.txt",
skip=62, nlines=2)
EosinophilsSalC10_1<-
scan("/Users/egoncal2/Work_Files/Data/Scripts/data_camundongos.txt",
skip=66, nlines=3)
EosinophilsSalC10_2<-
scan("/Users/egoncal2/Work_Files/Data/Scripts/data_camundongos.txt",
skip=71, nlines=1)
EosinophilsSOVCO1<-
scan("/Users/egoncal2/Work_Files/Data/Scripts/data_camundongos.txt",
skip=74, nlines=3)
EosinophilsSOVCO2<-
scan("/Users/egoncal2/Work_Files/Data/Scripts/data_camundongos.txt",
skip=79, nlines=2)

#eotaxin production in the peritoneal cavity
EotaxinSaline1<-
scan("/Users/egoncal2/Work_Files/Data/Scripts/data_camundongos.txt",
skip=85, nlines=2)
EotaxinSaline2<-
scan("/Users/egoncal2/Work_Files/Data/Scripts/data_camundongos.txt",
skip=89, nlines=1)
EotaxinSOACO1<-
scan("/Users/egoncal2/Work_Files/Data/Scripts/data_camundongos.txt",
skip=92, nlines=1)
EotaxinSOACO2<-
scan("/Users/egoncal2/Work_Files/Data/Scripts/data_camundongos.txt",
skip=95, nlines=1)
EotaxinS100C10_1<-
scan("/Users/egoncal2/Work_Files/Data/Scripts/data_camundongos.txt",
skip=98, nlines=2)
EotaxinS100C10_2<-
scan("/Users/egoncal2/Work_Files/Data/Scripts/data_camundongos.txt",
skip=102, nlines=2)

#Ant venom activates dendritic cells in vitro. (3 plots)
MHCIIeCD86.negCtr1<-
scan("/Users/egoncal2/Work_Files/Data/Scripts/data_camundongos.txt",
skip=108, nlines=4)
MHCIIeCD86.negCtr2<-
scan("/Users/egoncal2/Work_Files/Data/Scripts/data_camundongos.txt",
skip=114, nlines=2)
MHCIIeCD86.negCtr3<-
scan("/Users/egoncal2/Work_Files/Data/Scripts/data_camundongos.txt",
skip=118, nlines=2)
MHCIIeCD86.posCtr1<-
scan("/Users/egoncal2/Work_Files/Data/Scripts/data_camundongos.txt",
skip=122, nlines=4)
MHCIIeCD86.posCtr2<-
```

```
scan("/Users/egoncal2/Work_Files/Data/Scripts/data_camundongos.txt",
skip=128, nlines=2)
MHCIIeCD86.posCtr3<-
scan("/Users/egoncal2/Work_Files/Data/Scripts/data_camundongos.txt",
skip=132, nlines=2)
MHCIIeCD86V100_1<-
scan("/Users/egoncal2/Work_Files/Data/Scripts/data_camundongos.txt",
skip=136, nlines=4)
MHCIIeCD86V100_2<-
scan("/Users/egoncal2/Work_Files/Data/Scripts/data_camundongos.txt",
skip=142, nlines=2)
MHCIIeCD86V100_3<-
scan("/Users/egoncal2/Work_Files/Data/Scripts/data_camundongos.txt",
skip=146, nlines=2)
MHCIIeCD86V10_1<-
scan("/Users/egoncal2/Work_Files/Data/Scripts/data_camundongos.txt",
skip=150, nlines=4)
MHCIIeCD86V10_2<-
scan("/Users/egoncal2/Work_Files/Data/Scripts/data_camundongos.txt",
skip=156, nlines=2)
MHCIIeCD86V10_3<-
scan("/Users/egoncal2/Work_Files/Data/Scripts/data_camundongos.txt",
skip=160, nlines=2)
MHCIIeCD86V1_1<-
scan("/Users/egoncal2/Work_Files/Data/Scripts/data_camundongos.txt",
skip=164, nlines=4)
MHCIIeCD86V1_2<-
scan("/Users/egoncal2/Work_Files/Data/Scripts/data_camundongos.txt",
skip=170, nlines=2)
MHCIIeCD86V1_3<-
scan("/Users/egoncal2/Work_Files/Data/Scripts/data_camundongos.txt",
skip=174, nlines=2)
MHCIIeCD86V01_1<-
scan("/Users/egoncal2/Work_Files/Data/Scripts/data_camundongos.txt",
skip=178, nlines=4)
MHCIIeCD86V01_2<-
scan("/Users/egoncal2/Work_Files/Data/Scripts/data_camundongos.txt",
skip=184, nlines=2)
MHCIIeCD86V01_3<-
scan("/Users/egoncal2/Work_Files/Data/Scripts/data_camundongos.txt",
skip=188, nlines=2)
MHCIIineg.Ctr1<-
scan("/Users/egoncal2/Work_Files/Data/Scripts/data_camundongos.txt",
skip=194, nlines=4)
MHCIIineg.Ctr2<-
scan("/Users/egoncal2/Work_Files/Data/Scripts/data_camundongos.txt",
skip=200, nlines=2)
MHCIIineg.Ctr3<-
scan("/Users/egoncal2/Work_Files/Data/Scripts/data_camundongos.txt",
skip=204, nlines=2)
MHCIIipos.Ctr1<-
scan("/Users/egoncal2/Work_Files/Data/Scripts/data_camundongos.txt",
skip=208, nlines=4)
MHCIIipos.Ctr2<-
scan("/Users/egoncal2/Work_Files/Data/Scripts/data_camundongos.txt",
skip=214, nlines=2)
MHCIIipos.Ctr3<-
scan("/Users/egoncal2/Work_Files/Data/Scripts/data_camundongos.txt",
skip=218, nlines=2)
MHCIIiV100_1<-
```

```
scan("/Users/egoncal2/Work_Files/Data/Scripts/data_camundongos.txt",
skip=222, nlines=4)
MHCIIiV100_2<-
scan("/Users/egoncal2/Work_Files/Data/Scripts/data_camundongos.txt",
skip=228, nlines=2)
MHCIIiV100_3<-
scan("/Users/egoncal2/Work_Files/Data/Scripts/data_camundongos.txt",
skip=232, nlines=2)
MHCIIiV10_1<-
scan("/Users/egoncal2/Work_Files/Data/Scripts/data_camundongos.txt",
skip=236, nlines=4)
MHCIIiV10_2<-
scan("/Users/egoncal2/Work_Files/Data/Scripts/data_camundongos.txt",
skip=242, nlines=2)
MHCIIiV10_3<-
scan("/Users/egoncal2/Work_Files/Data/Scripts/data_camundongos.txt",
skip=246, nlines=2)
MHCIIiV1_1<-
scan("/Users/egoncal2/Work_Files/Data/Scripts/data_camundongos.txt",
skip=250, nlines=4)
MHCIIiV1_2<-
scan("/Users/egoncal2/Work_Files/Data/Scripts/data_camundongos.txt",
skip=256, nlines=2)
MHCIIiV1_3<-
scan("/Users/egoncal2/Work_Files/Data/Scripts/data_camundongos.txt",
skip=260, nlines=2)
MHCIIiV01_1<-
scan("/Users/egoncal2/Work_Files/Data/Scripts/data_camundongos.txt",
skip=264, nlines=4)
MHCIIiV01_2<-
scan("/Users/egoncal2/Work_Files/Data/Scripts/data_camundongos.txt",
skip=270, nlines=2)
MHCIIiV01_3<-
scan("/Users/egoncal2/Work_Files/Data/Scripts/data_camundongos.txt",
skip=274, nlines=2)
CD86eneg.Ctr1<-
scan("/Users/egoncal2/Work_Files/Data/Scripts/data_camundongos.txt",
skip=280, nlines=4)
CD86eneg.Ctr2<-
scan("/Users/egoncal2/Work_Files/Data/Scripts/data_camundongos.txt",
skip=286, nlines=2)
CD86eneg.Ctr3<-
scan("/Users/egoncal2/Work_Files/Data/Scripts/data_camundongos.txt",
skip=290, nlines=2)
CD86epos.Ctr1<-
scan("/Users/egoncal2/Work_Files/Data/Scripts/data_camundongos.txt",
skip=294, nlines=4)
CD86epos.Ctr2<-
scan("/Users/egoncal2/Work_Files/Data/Scripts/data_camundongos.txt",
skip=300, nlines=2)
CD86epos.Ctr3<-
scan("/Users/egoncal2/Work_Files/Data/Scripts/data_camundongos.txt",
skip=304, nlines=2)
CD86eV100_1<-
scan("/Users/egoncal2/Work_Files/Data/Scripts/data_camundongos.txt",
skip=308, nlines=4)
CD86eV100_2<-
scan("/Users/egoncal2/Work_Files/Data/Scripts/data_camundongos.txt",
skip=314, nlines=2)
CD86eV100_3<-
```

```
scan("/Users/egoncal2/Work_Files/Data/Scripts/data_camundongos.txt",
skip=318, nlines=2)
CD86eV10_1<-
scan("/Users/egoncal2/Work_Files/Data/Scripts/data_camundongos.txt",
skip=322, nlines=4)
CD86eV10_2<-
scan("/Users/egoncal2/Work_Files/Data/Scripts/data_camundongos.txt",
skip=328, nlines=2)
CD86eV10_3<-
scan("/Users/egoncal2/Work_Files/Data/Scripts/data_camundongos.txt",
skip=332, nlines=2)
CD86eV1_1<-
scan("/Users/egoncal2/Work_Files/Data/Scripts/data_camundongos.txt",
skip=336, nlines=4)
CD86eV1_2<-
scan("/Users/egoncal2/Work_Files/Data/Scripts/data_camundongos.txt",
skip=342, nlines=2)
CD86eV1_3<-
scan("/Users/egoncal2/Work_Files/Data/Scripts/data_camundongos.txt",
skip=346, nlines=2)
CD86eV01_1<-
scan("/Users/egoncal2/Work_Files/Data/Scripts/data_camundongos.txt",
skip=350, nlines=4)
CD86eV01_2<-
scan("/Users/egoncal2/Work_Files/Data/Scripts/data_camundongos.txt",
skip=356, nlines=2)
CD86eV01_3<-
scan("/Users/egoncal2/Work_Files/Data/Scripts/data_camundongos.txt",
skip=360, nlines=2)

#Footpad swelling demonstrating allergic reaction (2 figures)
Foot_swellSaline<-
read.table("/Users/egoncal2/Work_Files/Data/Scripts/data_camundongos.txt",
skip=366, nrows=5, sep="\t", header=TRUE,check.names = FALSE)
Foot_swellSOACO<-
read.table("/Users/egoncal2/Work_Files/Data/Scripts/data_camundongos.txt",
skip=374, nrows=5, sep="\t", header=TRUE,check.names = FALSE)
Foot_swellS10C10<-
read.table("/Users/egoncal2/Work_Files/Data/Scripts/data_camundongos.txt",
skip=382, nrows=5, sep="\t", header=TRUE,check.names = FALSE)
Foot_swellSOVCO<-
read.table("/Users/egoncal2/Work_Files/Data/Scripts/data_camundongos.txt",
skip=392, nrows=5, sep="\t", header=TRUE,check.names = FALSE)
Foot_swellSOCO<-
read.table("/Users/egoncal2/Work_Files/Data/Scripts/data_camundongos.txt",
skip=400, nrows=5, sep="\t", header=TRUE,check.names = FALSE)

#lymph node celullarity response demonstrating chemotaxy
CellsLNSaline1<-
scan("/Users/egoncal2/Work_Files/Data/Scripts/data_camundongos.txt",
skip=410, nlines=2)
CellsLNSaline2<-
scan("/Users/egoncal2/Work_Files/Data/Scripts/data_camundongos.txt",
skip=414, nlines=1)
CellsLNSaline3<-
scan("/Users/egoncal2/Work_Files/Data/Scripts/data_camundongos.txt",
skip=417, nlines=1)
CellsLNSOACO1<-
scan("/Users/egoncal2/Work_Files/Data/Scripts/data_camundongos.txt",
skip=420, nlines=3)
```

```
CellsLNSOACO2<-
scan("/Users/egoncal2/Work_Files/Data/Scripts/data_camundongos.txt",
skip=425, nlines=2)
CellsLNSOACO3<-
scan("/Users/egoncal2/Work_Files/Data/Scripts/data_camundongos.txt",
skip=429, nlines=1)
CellsLNS10_1<-
scan("/Users/egoncal2/Work_Files/Data/Scripts/data_camundongos.txt",
skip=432, nlines=3)
CellsLNS10_2<-
scan("/Users/egoncal2/Work_Files/Data/Scripts/data_camundongos.txt",
skip=437, nlines=2)
CellsLNS10_3<-
scan("/Users/egoncal2/Work_Files/Data/Scripts/data_camundongos.txt",
skip=441, nlines=1)
CellsLNS100_1<-
scan("/Users/egoncal2/Work_Files/Data/Scripts/data_camundongos.txt",
skip=444, nlines=3)
CellsLNS100_2<-
scan("/Users/egoncal2/Work_Files/Data/Scripts/data_camundongos.txt",
skip=449, nlines=2)
CellsLNS100_3<-
scan("/Users/egoncal2/Work_Files/Data/Scripts/data_camundongos.txt",
skip=453, nlines=1)

#cytokine response in lymph node cells, illustrated by IL-4
IL4Saline24h<-
scan("/Users/egoncal2/Work_Files/Data/Scripts/data_camundongos.txt",
skip=458, nlines=2)
IL4Saline48h<-
scan("/Users/egoncal2/Work_Files/Data/Scripts/data_camundongos.txt",
skip=462, nlines=2)
IL4OVA24h<-
scan("/Users/egoncal2/Work_Files/Data/Scripts/data_camundongos.txt",
skip=466, nlines=2)
IL4OVA48h<-
scan("/Users/egoncal2/Work_Files/Data/Scripts/data_camundongos.txt",
skip=470, nlines=2)
IL4S1024h<-
scan("/Users/egoncal2/Work_Files/Data/Scripts/data_camundongos.txt",
skip=474, nlines=2)
IL4S1048h<-
scan("/Users/egoncal2/Work_Files/Data/Scripts/data_camundongos.txt",
skip=478, nlines=2)
IL4S10024h<-
scan("/Users/egoncal2/Work_Files/Data/Scripts/data_camundongos.txt",
skip=482, nlines=2)
IL4S10048h<-
scan("/Users/egoncal2/Work_Files/Data/Scripts/data_camundongos.txt",
skip=486, nlines=2)

#Supplementary experiments on:
#####(i) inactivation of effect by heat;
Boiled_Neg_eosin<-
scan("/Users/egoncal2/Work_Files/Data/Scripts/data_camundongos.txt",
skip=493, nlines=4)
Boiled_Pos_eosin<-
scan("/Users/egoncal2/Work_Files/Data/Scripts/data_camundongos.txt",
skip=499, nlines=5)
Boiled_Boi_eosin<-
```

```
scan("/Users/egoncal2/Work_Files/Data/Scripts/data_camundongos.txt",
skip=507, nlines=6)
Boiled_Neg_eosin_total<-
scan("/Users/egoncal2/Work_Files/Data/Scripts/data_camundongos.txt",
skip=516, nlines=4)
Boiled_Pos_eosin_total<-
scan("/Users/egoncal2/Work_Files/Data/Scripts/data_camundongos.txt",
skip=522, nlines=6)
Boiled_Boi_eosin_total<-
scan("/Users/egoncal2/Work_Files/Data/Scripts/data_camundongos.txt",
skip=530, nlines=6)
#####(ii) PLA2 activity in venom
PLA2_Neg<-
read.table("/Users/egoncal2/Work_Files/Data/Scripts/data_camundongos.txt",
skip=540, nrows=3, sep="\t", header=FALSE,check.names = FALSE)
PLA2_Pos<-
read.table("/Users/egoncal2/Work_Files/Data/Scripts/data_camundongos.txt",
skip=546, nrows=3, sep="\t", header=FALSE,check.names = FALSE)
PLA2_Ven<-
read.table("/Users/egoncal2/Work_Files/Data/Scripts/data_camundongos.txt",
skip=552, nrows=3, sep="\t", header=FALSE,check.names = FALSE)

#### Formatting the data into long & summarised formats for plotting

#recruitment to peritoneal cavity following antigen exposure
#Grouping up the data into tables
EOsinophilsSaline<-c(EosinophilsSaline1, EosinophilsSaline2,
EosinophilsSaline3)
EOsinophilsSOACO<-c(EosinophilsSOACO1, EosinophilsSOACO2,
EosinophilsSOACO3)
EOsinophilsS10C10<-c(EosinophilsS10C10_1, EosinophilsS10C10_2,
EosinophilsS10C10_3)
EOsinophilsS100C10<-c(EosinophilsS100C10_1,EosinophilsS100C10_2,
EosinophilsS100C10_3)
EOsinophilsSalC10<-c(EosinophilsSalC10_1, EosinophilsSalC10_2)
EOsinophilsSOVCO<-c(EosinophilsSOVCO1, EosinophilsSOVCO2)
#Organising data converting to numbers
#Optimising dimensions names
Eosinophils<-mget(ls(pattern="Eosinophils"))
names(Eosinophils) <- sub("Eosinophils", "", names(Eosinophils))
EOsinophils<-mget(ls(pattern="EOsinophils"))
names(EOsinophils) <- sub("EOsinophils", "", names(EOsinophils))
#organising for plotting
long.Eosinophils<-melt(Eosinophils, id=Eosinophils$L1)
long.EOsinophils<-melt(EOsinophils, id=EOsinophils$L1)
#summarising data & adjusting plotting order with factors
summary.Eosinophils<-ddply(long.Eosinophils, "L1", summarise,
min=min(value, na.rm=T), max =max(value, na.rm=t),mean=mean(value,
na.rm=T), sd=sd(value, na.rm=T), se = sd/sqrt(length(value)))
summary.EOsinophils<-ddply(long.EOsinophils, "L1", summarise,
min=min(value, na.rm=T), max =max(value, na.rm=t),mean=mean(value,
na.rm=T), sd=sd(value, na.rm=T), se = sd/sqrt(length(value)))
#Adjusting factors orders to ggplot on x axis
summary.EOsinophils$L1<-factor(summary.EOsinophils$L1, levels=c("Saline",
"SOACO","S10C10", "S100C10","SOVCO", "SalC10"))
long.EOsinophils$L1<-factor(long.EOsinophils$L1, levels=c("Saline",
"SOACO","S10C10", "S100C10","SOVCO", "SalC10"))

#eotaxin production in the peritoneal cavity
#Grouping up the data into tables
```

```
EOtaxinSaline<-c(EotaxinSaline1, EotaxinSaline2)
EOtaxinSOACO<-c(EotaxinSOACO1, EotaxinSOACO2)
EOtaxinS100C10<-c(EotaxinS100C10_1, EotaxinS100C10_2)
##Organising data converting to numbers
#Optimising dimensions names
Eotaxin<-mget(ls(pattern="Eotaxin"))
names(Eotaxin) <- sub("Eotaxin", "", names(Eotaxin))
EOtaxin<-mget(ls(pattern="EOtaxin"))
names(EOtaxin) <- sub("EOtaxin", "", names(EOtaxin))
#organising for plotting
long.Eotaxin<-melt(Eotaxin, id=Eotaxin$L1)
long.EOtaxin<-melt(EOtaxin, id=EOtaxin$L1)
#summarising data & adjusting plotting order with factors
summary.Eotaxin<-ddply(long.Eotaxin, "L1", summarise, min=min(value,
na.rm=T), max =max(value, na.rm=t),mean=mean(value, na.rm=T), sd=sd(value,
na.rm=T), se = sd/sqrt(length(value)))
summary.EOtaxin<-ddply(long.EOtaxin, "L1", summarise, min=min(value,
na.rm=T), max =max(value, na.rm=t),mean=mean(value, na.rm=T), sd=sd(value,
na.rm=T), se = sd/sqrt(length(value)))
#Adjusting factors orders to ggplot on x axis
summary.EOtaxin$L1<-factor(summary.EOtaxin$L1,
levels=c("Saline","SOACO","S100C10"))
long.EOtaxin$L1<-factor(long.EOtaxin$L1,
levels=c("Saline","SOACO","S100C10"))


#Ant venom activates dendritic cells in vitro
#Grouping up the data into tables
MHCII_neg.Ctr<-c(MHCIIineg.Ctr1, MHCIIineg.Ctr2, MHCIIineg.Ctr3)
MHCII_pos.Ctr<-c(MHCIIipos.Ctr1, MHCIIipos.Ctr2, MHCIIipos.Ctr3)
MHCII_V100<-c(MHCIIiV100_1, MHCIIiV100_2, MHCIIiV100_3)
MHCII_V10<-c(MHCIIiV10_1, MHCIIiV10_2, MHCIIiV10_3)
MHCII_V1<-c(MHCIIiV1_1, MHCIIiV1_2, MHCIIiV1_3)
MHCII_V01<-c(MHCIIiV01_1, MHCIIiV01_2, MHCIIiV01_3)
MHCIIeCd86.NegCtr<-c(MHCIIeCD86.negCtr1, MHCIIeCD86.negCtr2,
MHCIIeCD86.negCtr3)
MHCIIeCd86.PosCtr<-c(MHCIIeCD86.posCtr1, MHCIIeCD86.posCtr2,
MHCIIeCD86.posCtr3)
MHCIIeCd86v100<-c(MHCIIeCD86V100_1, MHCIIeCD86V100_2, MHCIIeCD86V100_3)
MHCIIeCd86v10<-c(MHCIIeCD86V10_1, MHCIIeCD86V10_2, MHCIIeCD86V10_3)
MHCIIeCd86v1<-c(MHCIIeCD86V1_1, MHCIIeCD86V1_2, MHCIIeCD86V1_3)
MHCIIeCd86v01<-c(MHCIIeCD86V01_1, MHCIIeCD86V01_2, MHCIIeCD86V01_3)
CD86_neg.Ctr<-c(CD86eneg.Ctr1, CD86eneg.Ctr2, CD86eneg.Ctr3)
CD86_pos.Ctr<-c(CD86epos.Ctr1, CD86epos.Ctr2, CD86epos.Ctr3)
CD86_V100<-c(CD86eV100_1, CD86eV100_2, CD86eV100_3)
CD86_V10<-c(CD86eV10_1, CD86eV10_2, CD86eV10_3)
CD86_V1<-c(CD86eV1_1, CD86eV1_2, CD86eV1_3)
CD86_V01<-c(CD86eV01_1, CD86eV01_2, CD86eV01_3)
##Organising data converting to numbers
#Optimising dimensions names
MHCIIeCD86<-mget(ls(pattern="MHCIIeCD"))
names(MHCIIeCD86) <- sub("MHCIIeCD86", "", names(MHCIIeCD86))
MHCII<-mget(ls(pattern="MHCIIi"))
names(MHCII) <- sub("MHCII", "", names(MHCII))
CD86<-mget(ls(pattern="CD86e"))
names(CD86) <- sub("CD86", "", names(CD86))
#
MHCIIeCd86<-mget(ls(pattern="MHCIIeCd"))
names(MHCIIeCd86) <- sub("MHCIIeCd86", "", names(MHCIIeCd86))
MHCII_<-mget(ls(pattern="MHCII_"))
```

```
names(MHCII_) <- sub("MHCII_", "", names(MHCII_))
CD86_<-mget(ls(pattern="CD86_"))
names(CD86_) <- sub("CD86_", "", names(CD86_))
#
long.MHCIIeCD86<-melt(MHCIIeCD86, id=MHCIIeCD86$L1)
long.MHCIIeCd86<-melt(MHCIIeCd86, id=MHCIIeCd86$L1)
long.MHCII<-melt(MHCII, id=MHCII$L1)
long.MHCII_<-melt(MHCII_, id=MHCII_$L1)
long.CD86<-melt(CD86, id=CD86$L1)
long.CD86_<-melt(CD86_, id=CD86_$L1)
#summarising data & adjusting plotting order with factors
summary.MHCIIeCD86<-ddply(long.MHCIIeCD86, "L1", summarise, min=min(value,
na.rm=T), max =max(value, na.rm=t),mean=mean(value, na.rm=T), sd=sd(value,
na.rm=T), se = sd/sqrt(length(value)))
summary.MHCIIeCd86<-ddply(long.MHCIIeCd86, "L1", summarise, min=min(value,
na.rm=T), max =max(value, na.rm=t),mean=mean(value, na.rm=T), sd=sd(value,
na.rm=T), se = sd/sqrt(length(value)))
#
summary.MHCII<-ddply(long.MHCII, "L1", summarise, min=min(value, na.rm=T),
max =max(value, na.rm=t),mean=mean(value, na.rm=T), sd=sd(value, na.rm=T),
se = sd/sqrt(length(value)))
summary.MHCII_<-ddply(long.MHCII_, "L1", summarise, min=min(value,
na.rm=T), max =max(value, na.rm=t),mean=mean(value, na.rm=T), sd=sd(value,
na.rm=T), se = sd/sqrt(length(value)))
#
summary.CD86<-ddply(long.CD86, "L1", summarise, min=min(value, na.rm=T),
max =max(value, na.rm=t),mean=mean(value, na.rm=T), sd=sd(value, na.rm=T),
se = sd/sqrt(length(value)))
summary.CD86_<-ddply(long.CD86_, "L1", summarise, min=min(value, na.rm=T),
max =max(value, na.rm=t),mean=mean(value, na.rm=T), sd=sd(value, na.rm=T),
se = sd/sqrt(length(value)))
#
summary.MHCIIeCd86<-insertRow(summary.MHCIIeCd86,
c(1,0.0,0.0,0.0,0.0,0.0),3)
summary.MHCIIeCd86$L1<-
c("neg.Ctr","pos.Ctr","meupau","V0.1","V1.0","V10.0", "V100.0")
summary.MHCIIeCd86$L1<-factor(summary.MHCIIeCd86$L1,
levels=c("neg.Ctr","pos.Ctr","meupau", "V100.0","V10.0","V1.0","V0.1"))
summary.MHCII_<-insertRow(summary.MHCII_, c(1,0.0,0.0,0.0,0.0,0.0),5)

summary.MHCII_$L1<-c("V0.1","V1.0","V10.0",
"V100.0","meupau","neg.Ctr","pos.Ctr")
summary.MHCII_$L1<-factor(summary.MHCII_$L1,
levels=c("neg.Ctr","pos.Ctr","meupau", "V100.0","V10.0","V1.0","V0.1"))
summary.CD86_<-insertRow(summary.CD86_, c(1,0.0,0.0,0.0,0.0,0.0),5)
summary.CD86_$L1<-c("V0.1","V1.0","V10.0",
"V100.0","meupau","neg.Ctr","pos.Ctr")
summary.CD86_$L1<-factor(summary.CD86_$L1,
levels=c("neg.Ctr","pos.Ctr","meupau", "V100.0","V10.0","V1.0","V0.1"))

#Footpad data Fig. 5 & 6A
Foot_swell<-mget(ls(pattern="Foot_swell"))
names(Foot_swell) <- sub("Foot_swell", "", names(Foot_swell))
#organising for plotting
long.Foot_swell<-melt(Foot_swell)
##Organising data converting to numbers
#Optimising dimensions names
long.Foot_swell$variable<-gsub('.{1}$', '', long.Foot_swell$variable)
#
long.Foot_swell$L1<-factor(long.Foot_swell$variable, levels=c("Saline",
```

```
"SOACO", "S10C10", "SOVCO", "SOCO"))
#adjusting plotting order with factors
long.Foot_swell$Treat<-factor(long.Foot_swell$Treat, levels=c("Inoculated",
"30m", "60m", "90m", "120m"))
#summarising data
summary.Foot_swell<-ddply(long.Foot_swell, c("variable","Treat"),
summarise, min=min(value, na.rm=T), max =max(value,
na.rm=t),mean=mean(value, na.rm=T), sd=sd(value, na.rm=T), se =
sd/sqrt(length(value)))
#adjusting plotting order with factors
summary.Foot_swell$Treat<-factor(summary.Foot_swell$Treat,
levels=c("Inoculated", "30m", "60m", "90m", "120m"))


#Figure 7. Fire ant venom induces lymph node response.
#organising into tables
CEllsLNSaline<-c(CellsLNSaline1, CellsLNSaline2, CellsLNSaline3)
CEllsLNSOACO<-c(CellsLNSOACO1, CellsLNSOACO2, CellsLNSOACO3)
CEllsLNS10<-c(CellsLNS10_1, CellsLNS10_2, CellsLNS10_3)
CEllsLNS100<-c(CellsLNS100_1, CellsLNS100_2, CellsLNS100_3)
CellsLN<-mget(ls(pattern="CellsLN"))
CEllsLN<-mget(ls(pattern="CEllsLN"))
#setting up names
names(CEllsLN) <- sub("CEllsLN", "", names(CEllsLN))
names(CellsLN) <- sub("CellsLN", "", names(CellsLN))
#Making data into long format for plotting and adjusting plot order with
factors
long.CellsLN<-melt(CellsLN, id=CellsLN$L1)
long.CEllsLN<-melt(CEllsLN, id=CEllsLN$L1)
#
long.CellsLN$L1<-c(rep("S100",6), rep("S10",6), rep("SOACO", 6),
rep("Saline",4))
long.CellsLN$L1<-factor(long.CellsLN$L1, levels=c("Saline", "SOACO", "S10",
"S100"))
summary.CellsLN<-ddply(long.CellsLN, "L1", summarise, min=min(value,
na.rm=T), max =max(value, na.rm=t),mean=mean(value, na.rm=T), sd=sd(value,
na.rm=T), se = sd/sqrt(length(value)))
summary.CEllsLN<-ddply(long.CEllsLN, "L1", summarise, min=min(value,
na.rm=T), max =max(value, na.rm=t),mean=mean(value, na.rm=T), sd=sd(value,
na.rm=T), se = sd/sqrt(length(value)))
#adjusting plotting order with factors
summary.CEllsLN$L1<-factor(summary.CEllsLN$L1, levels=c("Saline", "SOACO",
"S10", "S100"))
long.CEllsLN$L1<-factor(long.CEllsLN$L1, levels=c("Saline", "SOACO", "S10",
"S100"))



#cytokine response in lymph node cells.
#making data into organised tables
Il424h<-data.frame(IL4Saline24h,IL4OVA24h,IL4S1024h,IL4S10024h)
Il448h<-data.frame(IL4Saline48h,IL4OVA48h,IL4S1048h,IL4S10048h)
#adjusting names
IL4<-mget(ls(pattern="IL4"))
names(IL4) <- sub("IL4", "", names(IL4))
#making into long format for plotting
long.IL4<-melt(IL4, id=IL4$L1)
long.Il424h<-melt(Il424h, id=IL4$L1)
long.Il448h<-melt(Il448h, id=IL4$L1)
#Adjusting names & plot order
```

```
long.IL4$L1<-sub("..h", "", long.IL4$L1)
long.IL4$L1<-factor(long.IL4$L1, levels=c("Saline", "OVA", "S10", "S100"))
#summarising numeric data
summary.IL4<-ddply(long.IL4, "L1", summarise, min=min(value, na.rm=T), max
=max(value, na.rm=t),mean=mean(value, na.rm=T), sd=sd(value, na.rm=T), se =
sd/sqrt(length(value)))
#adjusting plotting order with factors
summary.IL4$L1<-factor(summary.IL4$L1, levels=c("Saline", "OVA", "S10",
"S100"))


#Enzymatic adjuvant activity?
#Building tables for data with columns of uneven size (using spaces padded
with NA)
Boiled_eosin<-Filled.DataFrame(list(SOCO=Boiled_Neg_eosin,
SOVCO=Boiled_Pos_eosin, SOVhiCO=Boiled_Boi_eosin))
Boiled_eosin_total<-Filled.DataFrame(list(SOCO=Boiled_Neg_eosin_total,
SOVCO=Boiled_Pos_eosin_total, SOVhiCO=Boiled_Boi_eosin_total))
#summarising data but eliminating padded NAs
summary.Boiled_eosin_total<-ddply(melt(Boiled_eosin_total), "variable",
summarise, min=min(value, na.rm=T), max =max(value,
na.rm=t),mean=mean(value, na.rm=T), sd=sd(value, na.rm=T), se =
sd/sqrt(length(value)))
summary.Boiled_eosin<-ddply(melt(Boiled_eosin), "variable", summarise,
min=min(value, na.rm=T), max =max(value, na.rm=t),mean=mean(value,
na.rm=T), sd=sd(value, na.rm=T), se = sd/sqrt(length(value)))

#PLA2 activity in venom
#tables already done, so just making into long format; for some technical
reason, with no column names
long.PLA2_Neg<-melt(PLA2_Neg,id=PLA2_Neg$L1)
long.PLA2_Pos<-melt(PLA2_Pos,id=PLA2_Pos$L1)
long.PLA2_Ven<-melt(PLA2_Ven,id=PLA2_Ven$L1)
#summarising data without the first "zero" column
summary.PLA2_Neg<-ddply(long.PLA2_Neg[-c(1:3),], "variable", summarise,
min=min(value, na.rm=T), max =max(value, na.rm=t),mean=mean(value,
na.rm=T), sd=sd(value, na.rm=T), se = sd/sqrt(length(value)))
summary.PLA2_Pos<-ddply(long.PLA2_Pos[-c(1:3),], "variable", summarise,
min=min(value, na.rm=T), max =max(value, na.rm=t),mean=mean(value,
na.rm=T), sd=sd(value, na.rm=T), se = sd/sqrt(length(value)))
summary.PLA2_Ven<-ddply(long.PLA2_Ven[-c(1:3),], "variable", summarise,
min=min(value, na.rm=T), max =max(value, na.rm=t),mean=mean(value,
na.rm=T), sd=sd(value, na.rm=T), se = sd/sqrt(length(value)))
#turning into a summary table for plotting
PLA2<-rbind(summary.PLA2_Neg,summary.PLA2_Pos, summary.PLA2_Ven)
#adjusting names
PLA2$variable<-c(rep(c("0", "1", "5", "10", "15", "30", "45", "60"), 3))
PLA2$treat<-c(rep("NEG",8), rep("POS",8),rep("VEN",8))
#adjusting plotting order with factors
PLA2$treat<-factor(PLA2$treat, levels=c("NEG", "POS", "VEN"))
PLA2$variable<-factor(PLA2$variable, levels=c("0", "1", "5", "10", "15",
"30", "45", "60"))

#From this point all data is ready for analysis and plot

#Figure 2. "Ant venom promotes eosinophil recruitment to peritoneal cavity"
ggplot(long.EOsinophils, aes(x = L1, y = value, fill=(L1))) +
 geom_boxplot(width = 0.6, lwd = 1.5, fatten = 0.7)+
 scale_fill_hue(l = 45)+
 coord_cartesian(ylim = c(0, 40)) + theme_classic() +
```

```
 theme(legend.title = element_blank()) +
 labs(y="Eosinophils (% per cavity)\n", x = element_blank(), size = 40) +
 theme(axis.text.x = element_text(angle = 45, vjust = 0.6, size = 15)) +
 theme(axis.text.y = element_text(size = 15)) +
 theme(axis.title.y = element_text(size = 15)) +
 theme(legend.position="none") +
 scale_fill_manual(values=c("blue", "red", "gray", "gray", "gray", "gray"))


#Figure 3. Fire ant venom induces eotaxin production in the peritoneal
cavity.
#Option A: Boxplot. Problem is, there are not enough repetitions at the
moment
ggplot(long.EOtaxin, aes(x = L1, y = value, fill = (L1))) +
 geom_boxplot(width = 0.7, lwd = 1.5, fatten = 1) +
 scale_fill_hue(l = 45) +
 coord_cartesian(ylim = c(0, 800)) +
 theme_classic() +
 theme(legend.title = element_blank()) +
 labs(y = "Eotaxin (pg/mL)\n", x= element_blank(), size = 40) +
 theme(legend.position = "none") +
 theme(axis.text.y = element_text(size = 15)) +
 theme(axis.text.x = element_text(size = 15)) +
 theme(axis.title.y = element_text(size = 15)) +
 scale_fill_manual(values=c("blue", "red", "gray"))

#Option B: Column means plus raw values. Preferred in case we cannot amass
N>2 for SOACO

ggplot(NULL, aes(x = L1)) +
    geom_bar(data= summary.EOtaxin, stat = "identity", aes(y = mean),
width=0.6, fill = c("blue", "red", "gray"), colour="black")+
    geom_jitter(data=long.EOtaxin, aes(y=long.EOtaxin$value), width=0.1,
size=1)+
    labs(y = "Eotaxin (pg/mL)\n", x= element_blank(), size = 40) +
    scale_y_continuous(expand = c(0,0), limits = c(0,850))+
    theme_classic()


#Figure 4. Ant venom activates dendritic cells in vitro.
##MHCII
Fig.4A<- ggplot(data=summary.MHCII_, aes(x=L1, y=mean))+
geom_bar(stat="identity", width=c(0.8,0.8,0.8,0.8,0.8,0.8,0.8),
fill="#000000")+
geom_errorbar(aes(ymin= summary.MHCII_$mean-summary.MHCII_$se, ymax=
summary.MHCII_$mean+summary.MHCII_$se),position= position_dodge(width = 1),
width = c(0.2,0.2,0.2,0.2,0.2,0.2,0.2)) +
 theme_classic() +
 coord_cartesian(ylim = c(0, 30)) +
 theme(legend.title=element_blank())+
 labs(y="%MHCII Cells", x= "     Zymozan                      Venom
\u03BCg/mL")+
 scale_x_discrete(labels=c("-", "+", " ", "100.0", "10.0", "1.0", "0.1"))+
 scale_y_continuous(breaks=c(0,10,20,30), expand=c(0,0))+
 theme(axis.text.x = element_text(size=14),axis.text.y =
element_text(size=14), axis.title.y= element_text(size=18), axis.title.x=
element_text(hjust=-0.001, size=16))+
 ylab(expression("%"~MHCII^"\u002B"~Cells))
##CD86
Fig.4B<- ggplot(data=summary.CD86_, aes(x=L1, y=mean))+
```

```
geom_bar(stat="identity", width=c(0.8,0.8,0.8,0.8,0.8,0.8,0.8),
fill="#000000")+
geom_errorbar(aes(ymin= summary.CD86_$mean-summary.CD86_$se, ymax=
summary.CD86_$mean+summary.CD86_$se),position= position_dodge(width = 1),
width = c(0.2,0.2,0.2,0.2,0.2,0.2,0.2)) +
 theme_classic() +
 coord_cartesian(ylim = c(0, 50)) +
 theme(legend.title=element_blank())+
 labs(y="%CD86 Cells", x= "     Zymozan                         Venom
\u03BCg.mL")+
 scale_x_discrete(labels=c("-", "+", " ", "100.0", "10.0", "1.0", "0.1"))+
 scale_y_continuous(breaks=c(0,10,20,30, 40, 50), expand=c(0,0))+
 theme(axis.text.x = element_text(size=14),axis.text.y =
element_text(size=14), axis.title.y= element_text(size=18), axis.title.x=
element_text(hjust=-0.001, size=16))+
 ylab(expression("%"~CD86^"\u002B"~Cells))
#MHCII/CD86
Fig.4C<- ggplot(data=summary.MHCIIeCd86, aes(x=L1, y=mean))+
geom_bar(stat="identity", width=c(0.8,0.8,0.8,0.8,0.8,0.8,0.8),
fill="#000000")+
geom_errorbar(aes(ymin= summary.MHCIIeCd86$mean-summary.MHCIIeCd86$se,
ymax= summary.MHCIIeCd86$mean+summary.MHCIIeCd86$se),position=
position_dodge(width = 1), width = c(0.2,0.2,0.2,0.2,0.2,0.2,0.2)) +
 theme_classic() +
 coord_cartesian(ylim = c(0, 30)) +
 theme(legend.title=element_blank())+
 labs(y="%CD86/MHCII Cells", x= "     Zymozan                         Venom
\u03BCg/mL")+
 scale_x_discrete(labels=c("-", "+", " ", "100.0", "10.0", "1.0", "0.1"))+
 scale_y_continuous(breaks=c(0,10,20,30), expand=c(0,0))+
 theme(axis.text.x = element_text(size=14),axis.text.y =
element_text(size=14), axis.title.y= element_text(size=18), axis.title.x=
element_text(hjust=-0.001, size=16))+
 ylab(expression("%"~CD86/MHCII^"\u002B"~Cells))
##plotting composite figure
multiplot(Fig.4A, Fig.4B, Fig.4C)

#Figure 5. Footpad swelling after ant venom protein-fraction challenge.
Note: using SE
ggplot(summary.Foot_swell[(summary.Foot_swell$variable %in% c("SOACO",
"Saline", "S10C10")),],aes(x=Treat, y=mean, group=variable)) +
 coord_cartesian(ylim = c(0, 10)) +
 geom_line(aes(colour=variable), size=2) +
 theme_classic()+ theme (legend.position=c(0.15,0.85)) +
 scale_colour_manual(values=c("black", "red", "blue")) +
 geom_ribbon(aes(x=Treat, ymin=mean-se, ymax=mean+se, group=variable),
alpha=0.2) +
 xlab("Time post inoculation") +
 ylab(expression("Swelling in mm"^2)) +
 ggtitle("Footpad swelling following injection of fire ant venom") +
 theme(legend.title=element_blank())

#Figure 6. Adjuvant function of fire ant venom proteins. Note: using SE
ggplot(summary.Foot_swell[(summary.Foot_swell$variable %in% c("SOVCO",
"Saline", "SOCO")),],aes(x=Treat, y=mean, group=variable)) +
 coord_cartesian(ylim = c(0, 10))+ geom_line(aes(colour=variable), size=2)
+
 theme_classic()+ theme (legend.position=c(0.2,0.8)) +
 scale_colour_manual(values=c("black", "red", "blue")) +
 geom_ribbon(aes(x=Treat, ymin=mean-se, ymax=mean+se, group=variable),
```

```
alpha=0.2) +
 xlab("Time post inoculation") +
 ylab(expression("Swelling in mm"^2)) +
 ggtitle("Footpad swelling following injection of fire ant venom") +
 theme(legend.title=element_blank())

#Figure 7. Fire ant venom induces lymph node response.
ggplot(long.CEllsLN, aes(x = L1, y = value, fill=(L1))) +
 geom_boxplot(width = 0.7, lwd = 1.2, fatten = 1) +
 scale_fill_hue(l = 45) +
 coord_cartesian(ylim = c(0, 20)) +
 theme_classic() +
 theme(legend.title=element_blank()) +
 ylab(expression(sqrt(Cells~x~10^6~"/"~LN))) +
 theme(legend.position="none") +
 scale_fill_manual(values=c("blue", "red", "grey65", "grey35")) +
 theme(axis.text.y  = element_text(size=15)) +
 theme(axis.text.x  = element_text(size=15)) +
 theme(axis.title.y  = element_text(size=15)) +
 theme(axis.title.x  = element_blank())

#Figure 8. Ant venom induces cytokine response in lymph node cells.

par(mar=c(4,6,2,6))
barplot(-(as.numeric(lapply(Il424h,FUN=mean))), width=2.0, axis.lty = 1,
space=1, xlim=c(-30,30), ylim=c(2,50), horiz=T,
axisnames=T,col=c("blue","red","#B2AFAF","#434242"))
par(new=TRUE)
barplot((as.numeric(lapply(Il448h,FUN=mean))), width=2.0, axis.lty = 1,
space=1, xlim=c(-30,30), ylim=c(2,50), horiz=T,
axisnames=T,col=c("blue","red","#B2AFAF","#434242"))
legend(x="right", y="center", c("S10C100","S10C10", "OVA", "Saline"),
col=c("#434242","#B2AFAF","red", "blue"), lty="solid", lwd=10)
axis(2,pos=0,labels=F,col="black",lty=5, at=0:30, lwd.ticks=-1)
arrows(-((as.numeric(lapply(Il424h,FUN=max)))[2]),7, -
(as.numeric(lapply(Il424h,FUN=mean)))[2], lwd = 1, angle = 90,code = 3,
length = 0.05)
arrows(-((as.numeric(lapply(Il424h,FUN=max)))[3]),11, -
(as.numeric(lapply(Il424h,FUN=mean)))[3], lwd = 1, angle = 90,code = 3,
length = 0.05)
arrows(-((as.numeric(lapply(Il424h,FUN=max)))[4]),15, -
(as.numeric(lapply(Il424h,FUN=mean)))[4], lwd = 1, angle = 90,code = 3,
length = 0.05)
arrows(((as.numeric(lapply(Il448h,FUN=max)))[2]),7,
(as.numeric(lapply(Il448h,FUN=mean)))[2], lwd = 1, angle = 90,code = 3,
length = 0.05)
arrows(((as.numeric(lapply(Il448h,FUN=max)))[3]),11,
(as.numeric(lapply(Il448h,FUN=mean)))[3], lwd = 1, angle = 90,code = 3,
length = 0.05)
arrows(((as.numeric(lapply(Il448h,FUN=max)))[4]),15,
(as.numeric(lapply(Il448h,FUN=mean)))[4], lwd = 1, angle = 90,code = 3,
length = 0.05)
mtext(expression(IL4~(pg~"in"~10^6~Cells)), side=1, line=2.5, cex=1.1,
col="black")
mtext("24h   48h", side=1, line=-10.5, cex=1.5, col="black")
mtext("nd    nd", side=1, line=-1.85, cex=1.3,col="blue")

#Figure S2. Boiling fire ant venom proteins will inactivate innate
#Note a method for superposing plots from different datasets: feed the
first line with NULL data
```

```
ggplot(NULL, aes(x = variable)) +
    geom_boxplot(data= summary.Boiled_eosin, stat = "identity", aes(y= max,
ymin=mean-se, lower=mean-se, middle=mean, upper=mean+se, ymax=mean+se),
fatten = 0.1)+
    geom_jitter(data=melt(Boiled_eosin),aes(y=melt(Boiled_eosin)$value),
width=0.1, size=1)+
    scale_y_continuous(name="% eosinophils")+
    scale_x_discrete(name="Allergy treatment")+
    theme_classic()

#Figure S1. Phospholipase A2 activity from a venom protein extract of the
fire ant
ggplot(PLA2,aes(x=variable, y=mean, group=treat)) +
    coord_cartesian(ylim = c(0, 6)) +
 scale_x_discrete(expand = c(0,0))+
    geom_line(aes(colour=treat), size=1.3) +
    theme_classic() +
    theme (legend.position=c(0.5,0.8)) +
    geom_ribbon(aes(ymin=mean-se, ymax=mean+se), alpha=0.2) +
    xlab("Reaction Time (min)") +
    ylab("PLA Activity (\u03BCM/min)") +
    ggtitle("PLA2 in extracted fire ant venom protein") +
    theme(legend.title=element_blank())


#Statistical Analysis
#Numbers of repetitions too low to infer any assumptions about data
distribution (Normality or Homodisciestasy): Non-parametric tests are the
most reliable
#All treatments assume samples are independent (each individual is separate
from each other); two treatments should be compared by Wilcoxon Rank-Sum
(Mann-Whitney) and three treatments by Kruskal Walis; differences between
days by Kruskal Wallis
#dunn.test package is selected for multiple analysis for convenience of
interpretation

#require(dunn.test)#This version of Dunn Test is easy to interpret
#
#dunn.test(long.EOsinophils$value,long.EOsinophils$L1)
#  Kruskal-Wallis rank sum test
#
#data: x and group
#Kruskal-Wallis chi-squared = 23.09, df = 5, p-value = 0
#
#
#                           Comparison of x by group
#                                (No adjustment)
#Col Mean-|
#Row Mean |    S100C10     S10C10      SOACO      SOVCO     SalC10
#---------+-------------------------------------------------------
#  S10C10 |   0.298561
#         |     0.3826
#         |
#   SOACO |  -0.883899  -1.194661
#         |     0.1884     0.1161
#         |
#   SOVCO |  -0.333611  -0.590188   0.426983
#         |     0.3693     0.2775     0.3347
#         |
#  SalC10 |   1.553677   1.346270   2.226001   1.669185
```

```
#         |     0.0601     0.0891    0.0130*     0.0475
#         |
#  Saline |   3.434294   3.228869   4.196529   3.253616   1.200450
#         |    0.0003*    0.0006*    0.0000*    0.0006*     0.1150
#
#alpha = 0.05
#Reject Ho if p <= alpha/2
#
#>dunn.test(long.EOtaxin$value,long.EOtaxin$L1)
#  Kruskal-Wallis rank sum test
#
#data: x and group
#Kruskal-Wallis chi-squared = 5.8, df = 2, p-value = 0.06
#
#
#                           Comparison of x by group
#                                (No adjustment)
#Col Mean-|
#Row Mean |     Saline      SOACO
#---------+----------------------
#   SOACO |   2.200000
#         |     0.0139
#         |
# S100C10 |   1.912365  -0.632455
#         |     0.0279     0.2635
#
#
#
#dunn.test(long.MHCII_$value,long.MHCII_$L1)
#  Kruskal-Wallis rank sum test
#
#data: x and group
#Kruskal-Wallis chi-squared = 4.6187, df = 5, p-value = 0.46
#
#
#                           Comparison of x by group
#                                (No adjustment)
#Col Mean-|
#Row Mean |    neg.Ctr    pos.Ctr       V100        V10         V1
#---------+-------------------------------------------------------
# pos.Ctr |   1.392970
#         |     0.0818
#         |
#    V100 |   1.026869  -0.366101
#         |     0.1522     0.3571
#         |
#     V10 |   1.098303  -0.294666   0.071434
#         |     0.1360     0.3841     0.4715
#         |
#      V1 |   1.625132   0.232161   0.598262   0.526828
#         |     0.0521     0.4082     0.2748     0.2992
#         |
#     V01 |   1.982304   0.589333   0.955434   0.884000   0.357171
#         |     0.0237     0.2778     0.1697     0.1883     0.3605
#
#dunn.test(long.CD86_$value,long.CD86_$L1)
#  Kruskal-Wallis rank sum test
#
#data: x and group
#Kruskal-Wallis chi-squared = 12.5502, df = 5, p-value = 0.03
```

```
#
#
#                            Comparison of x by group
#                                 (No adjustment)
#Col Mean-|
#Row Mean |    neg.Ctr    pos.Ctr       V100        V10         V1
#---------+-------------------------------------------------------
# pos.Ctr |   3.393225
#         |     0.0003
#         |
#    V100 |   1.535881  -1.857344
#         |     0.0623     0.0316
#         |
#     V10 |   1.348360  -2.044864  -0.187520
#         |     0.0888     0.0204     0.4256
#         |
#      V1 |   1.964498  -1.428726   0.428617   0.616138
#         |     0.0247     0.0765     0.3341     0.2689
#         |
#     V01 |   2.259173  -1.134051   0.723292   0.910813   0.294674
#         |     0.0119     0.1284     0.2348     0.1812     0.3841
#
#
#dunn.test(long.MHCIIeCd86$value,long.MHCIIeCd86$L1)
#  Kruskal-Wallis rank sum test
#
#data: x and group
#Kruskal-Wallis chi-squared = 6.1841, df = 5, p-value = 0.29
#
#
#                            Comparison of x by group
#                                 (No adjustment)
#Col Mean-|
#Row Mean |    .NegCtr    .PosCtr        v01         v1        v10
#---------+-------------------------------------------------------
# .PosCtr |  -2.241558
#         |    0.0125*
#         |
#     v01 |  -1.911129   0.330428
#         |     0.0280     0.3705
#         |
#      v1 |  -1.652144   0.589413   0.258984
#         |     0.0493     0.2778     0.3978
#         |
#     v10 |  -1.125244   1.116313   0.785884   0.526900
#         |     0.1302     0.1321     0.2160     0.2991
#         |
#    v100 |  -1.214549   1.027008   0.696579   0.437595  -0.089305
#         |     0.1123     0.1522     0.2430     0.3308     0.4644
#
#alpha = 0.05
#Reject Ho if p <= alpha/2
#
#dunn.test(long.CEllsLN$value,long.CEllsLN$L1)
#  Kruskal-Wallis rank sum test
#
#data: x and group
#Kruskal-Wallis chi-squared = 15.0624, df = 3, p-value = 0
#
#
```

```
#                            Comparison of x by group
#                                 (No adjustment)
#Col Mean-|
#Row Mean |     Saline      SOACO        S10
#---------+---------------------------------
#   SOACO |   3.669093
#         |     0.0001
#         |
#     S10 |   1.541218  -2.379036
#         |     0.0616     0.0087
#         |
#    S100 |   1.242917  -2.712546  -0.333509
#         |     0.1069     0.0033     0.3694
#
##Statistics of PLA2 curves
#wilcox.test(long.PLA2_Neg$value, long.PLA2_Pos$value)
#
#Wilcoxon rank sum test with continuity correction
#
#data:  long.PLA2_Neg$value and long.PLA2_Pos$value
#W = 41.5, p-value = 2.411e-08
#alternative hypothesis: true location shift is not equal to 0
#
#wilcox.test(long.PLA2_Neg$value, long.PLA2_Ven$value)
#
# Wilcoxon rank sum test with continuity correction
#
#data:  long.PLA2_Neg$value and long.PLA2_Ven$value
#W = 43.5, p-value = 2.94e-08
#alternative hypothesis: true location shift is not equal to 0
#
#wilcox.test(long.PLA2_Pos$value, long.PLA2_Ven$value)
#
# Wilcoxon rank sum test with continuity correction
#
#data:  long.PLA2_Pos$value and long.PLA2_Ven$value
#W = 351.5, p-value = 0.8288
#alternative hypothesis: true location shift is not equal to 0
#
#dunn.test(melt(Boiled_eosin)$value,melt(Boiled_eosin)$variable)
#No id variables; using all as measure variables
#No id variables; using all as measure variables
#  Kruskal-Wallis rank sum test
#
#data: x and group
#Kruskal-Wallis chi-squared = 2.1733, df = 2, p-value = 0.34
#
#
#                            Comparison of x by group
#                                 (No adjustment)
#Col Mean-|
#Row Mean |     BOILED        NEG
#---------+----------------------
#     NEG |  -0.115470
#         |     0.4540
#         |
#     POS |  -1.378624  -1.133333
#         |     0.0840     0.1285
#
#BOILED IS NOT STATISTICALLY DIFFERENT FROM NEGATIVE CONTROL at alpha =
```

0.1, AND DIFFERED FROM POSITIVE CONTRO
